# Tractable nonlinear memory functions as a tool to capture and explain dynamical behaviors

**DOI:** 10.1103/PhysRevResearch.2.043069

**Published:** 2020-10-13

**Authors:** Edgar Herrera-Delgado, James Briscoe, Peter Sollich

**Affiliations:** The Francis Crick Institute, 1 Midland Rd., London NW1 1AT, United Kingdom and Department of Mathematics, King’s College London, Strand, London WC2R 2LS, United Kingdom; The Francis Crick Institute, 1 Midland Rd., London NW1 1AT, United Kingdom; Department of Mathematics, King’s College London, Strand, London WC2R 2LS, United Kingdom and Institut für Theoretische Physik, University of Göttingen, Friedrich-Hund-Platz 1, 37077 Göttingen, Germany

## Abstract

Mathematical approaches from dynamical systems theory are used in a range of fields. This includes biology where they are used to describe processes such as protein-protein interaction and gene regulatory networks. As such networks increase in size and complexity, detailed dynamical models become cumbersome, making them difficult to explore and decipher. This necessitates the application of simplifying and coarse graining techniques to derive explanatory insight. Here we demonstrate that Zwanzig-Mori projection methods can be used to arbitrarily reduce the dimensionality of dynamical networks while retaining their dynamical properties. We show that a systematic expansion around the quasi-steady-state approximation allows an explicit solution for memory functions without prior knowledge of the dynamics. The approach not only preserves the same steady states but also replicates the transients of the original system. The method correctly predicts the dynamics of multistable systems as well as networks producing sustained and damped oscillations. Applying the approach to a gene regulatory network from the vertebrate neural tube, a well-characterized developmental transcriptional network, identifies features of the regulatory network responsible for its characteristic transient behavior. Taken together, our analysis shows that this method is broadly applicable to multistable dynamical systems and offers a powerful and efficient approach for understanding their behavior.

## I Introduction

In complex dynamical systems, comprising multiple interacting components, it can be difficult to identify causal mechanisms and to dissect the function of parts of a system. Nonlinearities and feedback complicate intuitive understanding and these difficulties increase with the size and complexity of a system. Examples include biological processes such as protein-protein interaction and gene regulatory networks [1,2]. Mathematical models of these systems allow exploratory analysis and can provide insight but become less practical as system size grows. More importantly, the complexity can obscure the explanation for unexpected or emergent behaviors that originate in the dynamics of a system. For these reasons, a variety of approaches have been developed to reduce the complexity of models while pre-serving desired features of their behavior. An important class of tools are dimensionality-reduction techniques that coarse grain parts of a system [3–5].

The Zwanzig-Mori formalism provides an exact dimensionality reduction of a dynamical system based on a separation into an arbitrary subnetwork, the components of which are tracked explicitly, and a bulk containing the components that are replaced with memory functions [6–9]. These functions describe how the current subnetwork state feeds back, through the activity of molecular species in the bulk, to affect the subnetwork at a later time. This approach, specifically its nonlinear version, was originally developed for the dynamics of physical systems [10] but later generalized by Chorin *et al.* [11,12], with related uses also in optimal coarse graining [13]. A limitation, however, is that the memory functions are generally impossible to calculate in closed form [11,12]. Although approximate expressions can be derived in special cases [14–18], this restricts the applicability of the formalism. An alternative is to map the nonlinear system to a physical system consistent with the original; while this can be effective it does not necessarily simplify the problem [19,20]. Another option is to expand the dynamical equations around a fixed point and derive memory functions from this approximation [21,22], but for multistable or oscillatory systems the memory functions obtained in this way do not capture all the qualitative behaviors.

To address this limitation, we develop a method, based on the formalism of Ref. [11], that allows the calculation of memory functions for generic dynamical systems without prior knowledge of the dynamics. We make one assumption: the bulk must not generate fixed points beyond those of the sub-network, more specifically it must have a unique steady state for any subnetwork state, as assumed also in e.g. [18]. This is a natural condition: the subnetwork must be able to produce all fixed points itself, otherwise coarse graining cannot succeed. As the starting point for the dimensionality reduced dynamics we use the quasi-steady-state (QSS) approximation, where the bulk is always in steady state with the current subnetwork state as has been used in other contexts [23]. Memory functions are then constructed to correct the projected subnetwork state, by accounting for departures of the bulk from its steady state. Our main technical result is an explicit solution for the functions capturing these memory effects, derived in a systematic expansion around the QSS approximation. We demonstrate that the approach accurately predicts the dynamics of systems that produce multiple steady states and even sustained or damped oscillations. We also illustrate its use by applying it to a gene regulatory network from the embryonic vertebrate neural tube [24]. This is a transcriptional network of four interacting transcription factors with well described transient dynamics. We show how the memory functions generated by this approach provide insight into the features of the regulatory network that produce this transient behavior. Taken together, the analysis introduces a broadly applicable method for the investigation and analysis of complex dynamical systems.

## II Mathematical Derivation

### A Initial definitions

Following Ref. [11], we start from a system with degrees of freedom ***x*** evolving deterministically in time according to some nonlinear functions ***R***:

(1) $$\frac{dx}{dt}=R\left(x\right).$$

We define the flow ***φ***(***x***, *t*) as the state the system reaches at time *t* if it starts in some initial state ***x***; this function thus obeys ***φ***(***x***, 0) = ***x*** and $\frac{\partial }{\partial t}\phi \left(x,t\right)=R\left(\phi \left(x,t\right)\right)$. We want to understand the dynamics of some chosen set of observables that we denote by the vector ***A***. Such observables are functions of the state of the system, which we write as ***A***(***x***). By analogy with the definition of ***φ***, the time-dependent observables are then taken as

(2) A(x,t)=A(ϕ(x,t)),

so ***A***(***x***, *t*) gives the value of the observables at time *t* if the system was initially in state ***x***. The resulting time evolution of the observables can again be described by a differential equation

(3) $$\begin{array}{cc} \frac{\partial }{\partial t}A=LA\left(x\right) & L=\sum_{i}R_{i}\left(x\right) \end{array}\frac{\partial }{\partial x_{i}},$$

with the Liouvillian *L*, a linear differential operator. The general setup above requires us to track the full ***x*** dependence of the chosen observables ***A***(***x***, *t*). To achieve a reduction in dimensionality, Chorin [11] assumes that the value of *x* is determined by ***A*** at least statistically, i.e., has some probability distribution that (only) depends on the current ***A***. Averages (expectations) over this distribution are written as *E*[·|***A***], and the average evolution of ***A*** is governed by ***υ***(***A***) = *E*[*L**A***(·)|***A***]. Chorin [11,12] showed that the corrections to this in the actual time evolution take the form of a memory term and a so-called random force ***r***, giving the general form for the time evolution of ***A*** as

(4) $$\frac{d}{dt}A=\upsilon \left(A\right)+\int_{0}^{t}dt'M\left(A\left(t'\right),t-t'\right)+r.$$

The memory function ***M***(***A***(*t*'), *t* − *t*') depends on time difference *τ* = *t* − *t*' and—nonlinearly—on the past observable value ***A***(*t*'). Its evolution with *τ* is governed by the *deviations* of the drift from ***υ***(***A***); this evolution reads for a general observable *g*(***x***, *τ*):

(5) $$\frac{\partial }{\partial \tau }g\left(x,\tau \right)=Lg\left(x,t\right)-E\left[\left(Lg\right)\left(\cdot ,\tau \right)|A\left(x\right)\right].$$

The memory function is obtained from the observable that measures exactly such fuctuations in the drift of the observables ***A***:

(6) F(x)=(LA)(x)−E[LA(⋅)|A(x)].

We define an ***F***(***x***, *τ*) via Eq. (5) from the initial condition ***F***(***x***, 0) = ***F***(***x***), and the memory function is then given explicitly as ***M***(***A***, *τ*) = *E*[*L**F***(·, *τ*)|***A***]. The random force itself is ***r***(***x***, *t*) = ***F***(***x***, *t*) and has a vanishing average at all times, *E*[***r***(·, *t*)|***A***] = 0 [11]. In our context, this term represents effects that come from the bulk starting *away* from QSS. When the bulk starts in QSS, which is what we assume in the following, it vanishes and so can be discarded. While in Ref. [11] steady-state dynamics are discussed, this is not required for the above formalism to be applicable.

### B Subnetwork dynamics

With the random force discarded as above, Eq. (4) is a closed equation for the time evolution of the observables ***A*** and so achieves the desired dimensionality reduction. However, the memory function cannot in general be calculated in any closed form. We now show that this *can* be done within a systematic approximation for subnetwork dynamics. By this, we mean that we consider as the observables ***A*** = ***x***^s^ a subset of ***x***, e.g., the concentrations of molecular species in a subnetwork of a large gene regulatory network. We denote the degrees of freedom in the rest of the network, the bulk, by ***x***^b^ and write out the components of the general time evolution Eq. (1) as

(7) $$\begin{array}{cc} \frac{dx^{\text{s}}}{dt}=R^{\text{s}}\left(x^{\text{s}},x^{\text{b}}\right), & \frac{dx^{\text{b}}}{dt} \end{array}=R^{\text{b}}\left(x^{\text{s}},x^{\text{b}}\right).$$

The Liouvillian then splits accordingly into

(8) $$L=\sum_{s}R_{s}\left(x^{\text{s}},x^{\text{b}}\right)\frac{\partial }{\partial x_{s}}+\sum_{b}R_{b}\left(x^{\text{s}},x^{\text{b}}\right)\frac{\partial }{\partial x_{b}},$$

with the sums running over subnetwork and bulk species, respectively. Here and below, subscripts always indicate individual species, while vectors with s and b superscripts collect all subnetwork and bulk quantities, respectively. With Eq. (8), the generic observable time evolution of Eq. (3) (*∂/∂t*) ***x***^s^ = ***Lx***^s^ reduces to Eqs. (7) as it should. We now need to choose how to define the expectation *E*[·| ***x***^s^]. We do this so that, without the memory kernel, the reduced Eq. (4) corresponds to the simplification where the bulk dynamics equilibrates rapidly to any prevailing subnetwork state ***x***^s^, reaching a QSS value ***x***^b^∗ defined by *d**x***^b^/*dt* = 0 or

(9) $$R^{\text{b}}\left(x^{\text{s}},x^{\text{b}*}\left(x^{\text{s}}\right)\right)=0$$

As motivated in the Introduction, we will assume that this condition determines a unique bulk QSS ***x***^b^∗(***x***^s^) for any ***x***^s^. The expectation required to construct the reduced Eq. (4) is taken accordingly as

(10) $$E\left[g\left(\cdot \right)|x^{\text{s}}\right]=g\left(x^{\text{s}},x^{\text{b}*}\left(x^{\text{s}}\right)\right),$$

i.e., by taking ***x***^s^ as prescribed and inserting for ***x***^b^ its QSS value. The average drift ***υ***(***A***) = *E*[*L**A***(·)|***A***] = *E*[***R***^s^(***x***^s^, ***x***^b^)|***x***^s^] now evaluates directly from Eq. (10) as

(11) $$\upsilon \left(x^{\text{s}}\right)=R^{\text{s}}\left(x^{\text{s}},x^{\text{b}*}\left(x^{\text{s}}\right)\right).$$

This is the QSS or fast bulk approximation to the subnetwork dynamics. Our main interest in the following lies in understanding the memory effects that account for the fact that the bulk is not in general fast, but evolves on a timescale comparable to that of the subnetwork. To determine the resulting memory function, we start from the definition of ***F***(***x***), which from Eq. (6) has components

(12) $$F_{s}\left(x^{\text{s}},x^{\text{b}}\right)=R_{s}\left(x^{\text{s}},x^{\text{b}}\right)-R_{s}\left(x^{\text{s}},x^{\text{b}*}\left(x^{\text{s}}\right)\right).$$

The main challenge is now to calculate the evolution of this observable in time according to Eq. (5). This is not feasible in general but we can develop a systematic approximation by *linearizing* in deviations of the bulk degrees of freedom from the QSS, which we write as

(13) $$F_{s}\left(x^{\text{s}},x^{\text{b}},\tau \right)\approx \sum_{b}\left(x_{b}-x_{b}^{*}\left(x^{\text{s}}\right)\right)f_{bs}\left(x^{\text{s}},\tau \right).$$

The problem then reduces to finding the evolution of *f_bs_*(***x***^s^, *τ*) from the initial condition $f_{bs}\left(x^{s},0\right)\equiv f_{bs}^{0}\left(x^{s}\right)$ obtained by linearizing Eq. (12),

(14) $$f_{bs}^{0}\left(x^{\text{s}}\right)=\frac{\partial R_{s}}{\partial x_{b}},$$

where the derivatives here and below are evaluated at (***x***^s^, ***x***^b^∗(***x***^s^)) unless otherwise specified.

### C Memory evolution over time

To derive our memory function, we insert Eq. (13) into Eq. (5). Consistently applying the linearization as detailed in Appendix A yields the following equation for *f_bs_*:

(15) $$\frac{\partial }{\partial \tau }f_{bs}=\sum_{b'}l_{bb'}f_{b's}+\sum_{s'}R_{s'}\frac{\partial }{\partial x_{s'}}f_{bs}$$

with

(16) $$l_{bb'}=J_{b'b}+\sum_{s'b"}{\left(J^{-1}\right)}_{b'b"}\frac{\partial R_{b"}}{\partial x_{s'}}\frac{\partial R_{s'}}{\partial x_{b}},$$

where the Jacobian matrix ***J*** is defined as

(17) $$J_{b"b'}=\frac{\partial R_{b"}}{\partial x_{b'}}.$$

The next step is to find a solution *f_bs_*(***x***^s^, *τ*) for the partial differential equation given in Eq. (15). This can be done using the method of characteristics as the equation is linear in *f_bs_*(***x***^s^, *τ*) and only involves first derivatives, and gives the closed form solution (see Appendix B)

(18) $$f_{bs}\left(x^{\text{s}},\tau \right)=\sum_{b'}E_{bb'}\left(\tau \right)f_{b's}^{0}\left(\phi_{\upsilon }\left(x^{\text{s}},\tau \right)\right).$$

Here the *E*_*bb*'_ are elements of the time-ordered matrix exponential $E\left(\tau \right)=\exp\left[\int_{0}^{\tau }d\tau 'l\left(\phi_{\upsilon }\left(x^{\text{s}},\tau '\right)\right)\right]$ and the propagation in time is performed with the flow *φ_υ_* for the QSS drift ***υ***(***x***^s^).

### D Memory function

We can now finally determine the memory function on subnetwork species *s*, which from the general framework set out above is

(19) $$M_{s}\left(x^{\text{s}},\tau \right)=E\left[LF_{s}\left(\cdot ,\tau \right)|x^{\text{s}}\right].$$

We insert the expansion Eq. (13) here and obtain after some algebra (see Appendix A) our main result, a simple expression for the memory function,

(20) $$M_{s}\left(x^{\text{s}},\tau \right)=\sum_{b'}c_{b'}\left(x^{\text{s}}\right)f_{b's}\left(x^{\text{s}},\tau \right),$$

where we have denoted

(21) $$c_{b'}\left(x^{\text{s}}\right)=\sum_{s'}\sum_{b"}{\left(J^{-1}\right)}_{b'b"}\frac{\partial R_{b"}}{\partial x_{s'}}R_{s'}.$$

These functions can be thought of as prefactors to the memory term. The general projected time evolution equation now takes the form

(22) $$\frac{d}{dt}x_{s}=\upsilon \left(x^{\text{s}}\left(t\right)\right)+\int_{0}^{t}dt'M_{s}\left(x^{\text{s}}\left(t'\right),t-t'\right).$$

The first term contains the QSS drift while the second one represents the memory correction to this, which is expressed in terms of the memory function Eq. (20). Our derivation allows this memory to cover the behavior around multiple fixed points of the system, due to its nonlinear dependence on ***x***^s^. The interpretation of our result Eq. (20) is that in a small time interval $x_{b}-x_{b}^{*}$ will change by *c*(***x***^s^(*t*')) *dt*'. This deviation from the QSS is propagated by the exponential matrix and affects the drift *R_s_* at time *t* as captured by $f_{bs}^{0}$ in Eq. (18). In Appendix H, we compare Eq. (20) with the work of Ref. [18], which instead of the QSS assumption takes ***x***^b^ = 0. This is unsuitable for the multistable systems we are interested in but we show that the method can be adapted to project to bulk QSS values (Appendix I). This leads to an expression similar to Eq. (20), but crucially without the propagation in time from *t*' to *t* = *t*' + *τ* (Appendix I).

### E Self-consistent approximation

Our linearization approach Eq. (13) implies that the memory term captures dynamical effects that are of first order in the deviations of the bulk network from its QSS. We will now develop an approximate self-consistent way of incorporating higher order corrections, which turns out also to simplify the numerical evaluation of the memory terms. Consider the factor $f_{bs}^{0}\left(\phi_{\upsilon }\left(x^{\text{s}},\tau \right)\right)$ that from Eqs. (18) and (20) appears in the memory function *M_s_*(***x***^s^, *τ*). In the actual memory integral this is evaluated for ***x***^s^(*t*') and *τ* = *t* − *t*', i.e., as $f_{bs}^{0}\left(\phi_{\upsilon }\left(x^{s}\left(t'\right),t-t'\right)\right)$. As explained above, *φ_υ_* is the flow generated only by the QSS drift, i.e., without memory corrections. But the memory terms change the flow, so we can make the approach self-consistent by substituting for *φ_υ_* the actual time evolution *with* memory. This corresponds to replacing

(23) $$\phi_{\upsilon }\left(x^{s}\left(t'\right).t-t'\right)\to x^{\text{s}}\left(t\right),$$

as we are just propagating the subnetwork state from ***x***^s^(*t*') by a time difference *t* − *t*' to ***x***^s^(*t*). Making this replacement also in the matrix exponential in Eq. (18) changes the memory term $M_{s}\left(t\right)=\int_{0}^{t}dt'M_{s}\left(x^{\text{s}}\left(t'\right),t-t'\right)$ into

(24) $$\begin{array}{c} {\tilde{M}}_{s}\left(t\right)=\sum_{b"}\int_{0}^{t}dt'\sum_{b'}c_{b'}\left(x^{\text{s}}\left(t'\right)\right){\left(e^{\int_{t'}^{t}dt"l\left(x^{\text{s}}\left(t"\right)\right)}\right)}_{b'b"}\\ \times f_{b"s}^{0}\left(x^{\text{s}}\left(t\right)\right). \end{array}$$

The dependence on the subnetwork species *s* on which the memory acts is contained only in the—now *t*' independent— factor $f_{b",s}^{0}\left(x^{\text{s}}\left(t\right)\right)$. As shown in Appendix C, the memory integrals in the first line can then be calculated efficiently as solutions to differential equations, one for each bulk species *b*″. Conceptually, however, the self-consistent memory term is more complicated. In the original formulation Eq. (22), the memory is a superposition of separate effects from all past times *t*' : the state ***x***^s^(*t*') of the subnetwork affects the behavior of the bulk and feeds back into the subnetwork at time *t*. In Eq. (24), the way this feedback acts is additionally modulated by the entire time evolution of the subnetwork between times *t*' and *t*. In the applications considered below, both approaches yield similar quantitative results, hence which one to choose depends on the aim: for numerical calculations of memory effects, the self-consistent version is more efficient, whereas the memory functions themselves are easier to analyze in the original version because they depend—in addition to time difference, which always features—only on the subnetwork state at one time *t*'. Note that what we refer to as self-consistency is distinct from an approach widely used in the application of projection methods to physical systems (see, e.g., Refs. [25,26]), where equations of motion for correlation functions are considered and the relevant memory kernels are, via appropriate approximations, related back self-consistently to the correlation functions.

### F General memory properties

Both the ZMn and ZMs expressions for the memory term that we have derived are, as we have emphasized, nonlinear in ***x***^s^ and so not of the convolution form that would appear in linear ZM projection methods. This simpler form is recovered, however, in the dynamics near fixed points. To see this, we note that at a global fixed point (***x***^s^∗, ***x***^b^∗), where *R_s_* = 0, the last factor in the definition Eq. (21) of *c*_b'_ vanishes. As *c_b_* is a factor in both of our memory expressions, both receive zero contributions when ***x***^s^(*t*') is at a fixed point. Assuming *R_s_* and *R_b_* are sufficiently smooth, we can therefore *linearize* the memory terms for dynamics near such a fixed point. In this linearization, all other factors in the memory are evaluated at the fixed point, and from this one deduces (see Appendix G) that the linearized forms of the ZMn and ZMs memory expressions are in fact identical. The memory terms then become time convolutions of ***x***^s^(*t*') − ***x***^s^∗ with a memory kernel that depends only on time; Appendix G provides a numerical example. It follows from the general arguments in Appendix F that these kernels describe *exactly* the dynamics of the full linearized system, and so the equations with memory will correctly predict, e.g., the relaxation behavior near a stable fixed point.

### G Memory decomposition

In spite of their nonlinearity, it turns out to be possible to decompose our memory expressions into specific channels to analyze the contribution of interactions within a network. Building on the approach of Ref. [27], we take advantage of the two partial derivative expressions in Eqs. (14) and (21) to decompose the memory exactly into combinations of incoming and outgoing channels (Appendix D). The analogous construction for the self-consistent approximation is set out in Appendix E.

## III Applications

To test the effectiveness of the method, we examine systems that contain multiple steady states, oscillatory behaviors, and complex transient dynamics. These are relevant in a wide range of physical and biological contexts.

### A Multistability

We first examine a series of multistable systems defined by mutually repressive Hill functions:

(25) $$\frac{d}{dt}x_{j}=\frac{a}{1+\sum_{i\neq j}x_{i}^{n}}-x_{j}.$$

The above equation constitutes an “or” logic because of the sum of the terms in the denominator, where even if only one repressor has a high concentration, the production rate will become very low. Such interactions lead to multistability in a wide variety of developmental systems [28].

**FIG. 1:**
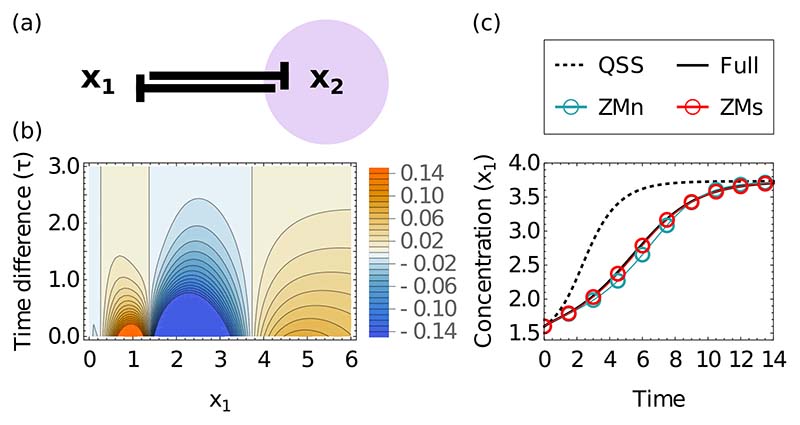
(a) Network illustrating a bistable switch defined using cross-repressive Hill functions Eq. (25) with *a* = 4, *n* = 2 [steady states are (*x*_1_, *x*_2_) = (6, 0.028) and (0.028, 6) with an unstable fixed point at (1.46,1.46)]. For this and all other network illustrations, blunt arrows indicate repression; purple shading identifies the species placed in the bulk. (b) Memory function for the bistable switch shown as a function of (past) concentration *x*_1_ of species 1 (*x* axis) and time difference *τ* (*y* axis). Memory function values range from negative to positive as indicated by the scale bar on the right and are capped by blue and orange outside the scale bar range. (c) Time course of the system demonstrates the capacity of both the nonlinear (ZMn, cyan) and self-consistent (ZMs, red) projections to capture the timescale and shape of transients of the full model (solid line) in reaching a stable fixed point. The QSS approximation (dashed line) significantly underestimates the length of the transient, showing that the ZM projections successfully correct for *x*_2_ not being at QSS.

We test the method on the simplest case with two nodes {*x*_1_, *x*_2_}, which leads to a system that cannot produce oscillations [29]. We place *x*_2_ in the bulk and calculate the memory function for the single remaining subnetwork species *x*_1_ [Fig. 1(a)]. This depends on the past concentration *x*_1_(*t*') and the time difference *τ* = *t* − *t*' [Fig. 1(b)]. We observe that the memory becomes zero at each fixed point as expected from the discussion in Sec. II F above and from Ref. [27], where the memory was obtained as an expansion (to quadratic order) in deviations of ***x***^s^ from a fixed point. To leading order, the memory grows linearly with this deviation, and in line with this we see it changing sign at every fixed point. The sign of the memory in all cases is opposite to that of the drift, so the memory delays the relaxation time to the corresponding steady state. This makes intuitive sense as in the original system, the bulk species’ state reacts relatively slowly to subnetwork changes, rather than infinitely fast as the QSS approximation assumes.

To test the accuracy of our method in capturing the transient temporal dynamics, we set the initial condition of *x*_1_ to be close to the unstable fixed point of the QSS dynamics; here we are furthest from the stable fixed points and so can test the limits of the method. For the evaluation, we used as a baseline the full dynamics of the original system, setting *x*_2_ at time zero to its QSS value with respect to the value of *x*_1_. We compare this to the subnetwork dynamics predicted by the simple QSS dynamics, and by our approach, which includes memory corrections. For this example, we evaluate both the nonlinear memory description Eq. (22), ZMn, and the self-consistent memory Eq. (24), denoted ZMs below. We find that both replicate the behavior of the original system well, independently of whether the initial condition eventually leads to the low- or high-*x*_1_ fixed point. The QSS approximation, on the other hand, reaches the steady state unrealistically fast [Fig. 1(c)]. Given that the ZMs method is substantially easier to implement for time course prediction (Appendix C), we concentrate on this approach below. Further justification for this comes from the fact that the self-consistent memory description is *exact* when *R*^s^ and *R*^b^ depend at most linearly on the bulk species, as we show in Appendix F. This exactness is not a trivial consequence of the fact that our approach is a linearization in ***x***^b^ − ***x***^b^∗, as it would otherwise hold also in the ZMn version. Biochemical systems with linear ***x***^b^ dependencies usually involve mass-action reactions and can produce bistable systems or oscillations [30,31], which we can then reproduce exactly with the ZMs projection (see Appendix F). A well-known example from physics is the Caldeira-Leggett model of a heat bath. This has a bulk composed of harmonic oscillators [32], and the resulting memory term was obtained exactly by Zwanzig [10,33]. Since in that system the bulk degrees of freedom appear linearly, our approach reproduces this solution but is more general in that it does not, for example, rely on the dynamics to be derived from a Hamiltonian.

**FIG. 2:**
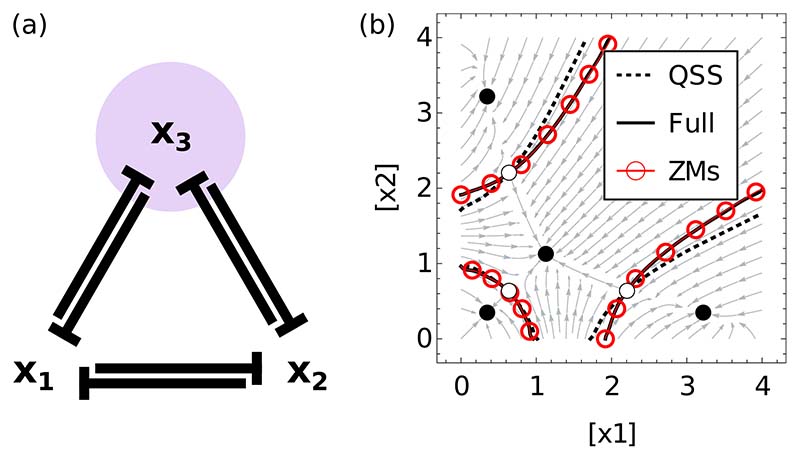
(a) Network illustrating the cross-repressive tetrastable system Eq. (25); purple shading indicates the species placed in the bulk. Parameters are *a* = 4, *n* = 2. (b) Phase portrait indicating the basins of attraction of the four stable fixed points (black circles) and the unstable fixed points (white circles). The separatrices bounding each basin are shown for the full dynamics (solid lines), QSS approximation (dotted lines), and the subnetwork equations with memory (ZMs, red circles); stream plots are shown for the QSS approximation. The QSS approach shows a clear difference to the original system, while the boundaries set by ZMs and the full system are almost indistinguishable.

We next tested the approach on a tetrastable system defined in the same way as Eq. (25) with variables {*x*_1_, *x*_2_, *x*_3_}. We consider the subnetwork containing *x*_1_ and *x*_2_, which will allow us to investigate the effect of the memory effects on the shapes of the basins of attraction of the different (stable) fixed points [Fig. 2(a)]. For the parameter values we use, there are four such fixed points for the full network: three where only one species has high concentration and the other two low, and one where all concentrations are equal [Fig. 2(b)]. The boundaries of the basins of attraction can be read as the points where, depending on its initial condition, the system chooses a different basin of attraction. In a biological setting, these choices could represent cell fate decisions, where a cell decides to adopt a specific specialized function and become a particular cell type (for a review, see Ref. [34]). We find that the QSS system fails to replicate the decision process of the original system, whereas the ZMs accurately identifies both the eventual steady state [Fig. 2(b)] *and* the timing to get to this state (data not shown).

### B Oscillations

**FIG. 3:**
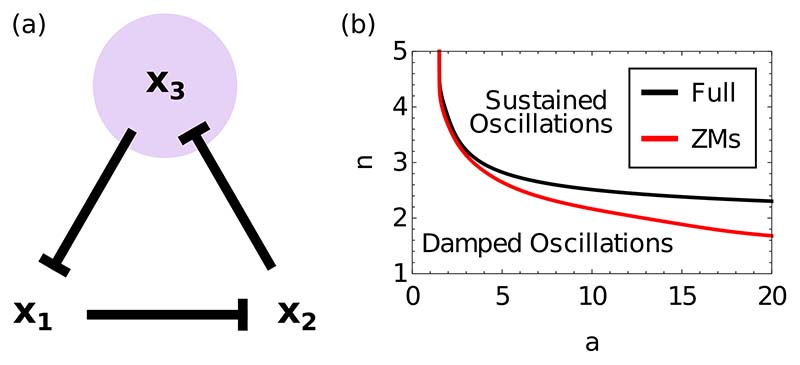
(a) Network illustrating the repressilator system Eq. (26); purple shading indicates the species placed in the bulk. (b) Bifurcation diagram of the repressilator for system parameters *a* and *n*. The lines represent supercritical Hopf bifurcations. The QSS system can only produce damped oscillations, so has no bifurcation at all. The ZMs system (red line) shows a good qualitative match to the shape and position of the bifurcation of the full system (solid black line).

We further explore the ability of the subnetwork equations with memory to reproduce oscillations arising from a unidirectional repressive network. We use the repressilator system [35], which robustly generates oscillations due to delays arising from an odd number of nodes [29,36]. It has concentration variables {*x*_1_, *x*_2_, *x*_3_} and repressive interactions as shown in Fig. 3(a) and represented mathematically by

(26) $$\frac{d}{dt}x_{j}=\frac{a}{1+x_{j-1}^{n}}-x_{j},$$

where *x*_0_ ≡ *x*_3_. We first compare the bifurcation diagram that results from varying both system parameters *a* and *n*, in order to see whether the subnetwork equations with memory can replicate the 2D Hopf bifurcation of the original system, from damped to sustained oscillations [Fig. 3(b)].

**FIG. 4:**
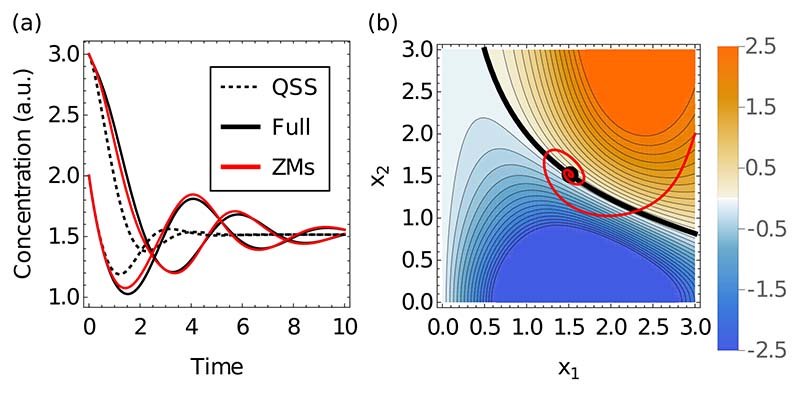
(a) Damped oscillations generated by the repressilator [35] can be accurately reproduced by the projection approach (red line) while the QSS (dotted line) fails to replicate both the timing and the amplitude of the oscillations of the full system (solid black line). (b) Color map of memory amplitude (memory at *τ* = 0) as a function of (*x*_1_, *x*_2_). The memory amplitude changes from negative (blue) to positive (orange) across the thick black line. Red curve: Parametric plot of the ZMs time course from the left; the memory repeatedly changes from negative to positive to drive the correct oscillations.

In contrast to the QSS approximation, we find that the projection technique correctly replicates the existence of sustained oscillations and predicts a qualitatively correct bifurcation diagram. The period and amplitude of sustained oscilations in the relevant parameter regime is less well replicated (not shown). For damped oscillations, the subnetwork equations with memory work accurately in predicting the full temporal dynamics [Fig. 4(a)]. By contrast, the QSS approximation displays almost no oscillatory behavior and none of a sustained nature, highlighting the importance of memory effects for oscillatory transients.

To understand in more detail how memory generates oscillations, we analyze the corresponding memory functions. We first plot the memory amplitude, i.e., the value *M*(***x***^s^, 0) for memory from the immediate past (*t*' = *t*) across the configuration space of our subnetwork [Fig. 4(b)] and observe two distinct regions with positive and negative memory amplitude, separated by a line where this amplitude vanishes (black). Plotting the time course from Fig. 4(a) in the same representation, we observe that it crosses the black line many times. The corresponding changes in the sign of the memory amplitude are what drives the oscillations seen in Fig. 4(a).

## IV Neural Tube Network (Transients and Multistability)

**FIG. 5:**
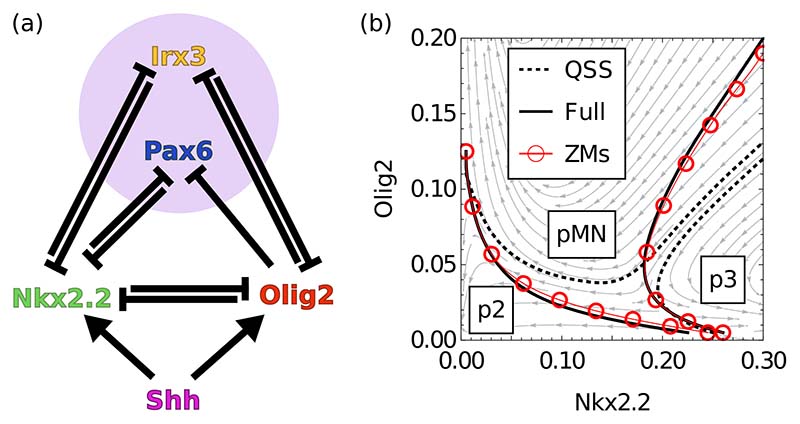
(a) Neural tube network [24] defined by cross-repressive interactions between four transcription factors and activation by Sonic Hedgehog signaling (Shh), dependent on neural tube position; purple shading indicates choice of bulk. (b) Fate decision diagram for a neural tube position with three attractors (at position *p* = 0.65); solid lines indicate boundaries of basins of attraction that biologically separate different fate choices. Possible steady states are p3 (high Nkx2.2, right), pMN (high Olig2, top), and p2 (low Nkx2.2 and Olig2, and high Irx3 and Pax6, bottom left). Dashed lines indicate basin boundaries for the QSS approximation and red dots the basin boundaries for the ZMs projection; a stream plot is shown for the QSS system. The ZMs system very accurately reproduces the boundaries of the full system.

Finally, we apply the projection approach to a biologically relevant system with several bifurcations and nontrivial dynamical properties, specifically the neural tube gene regulation network described in Ref. [24]. The network is sketched in Fig. 5(a) and its specific form and parametrization is given in Appendix J. In each cell of the neural tube, the network responds to the concentration of an extracellular signaling molecule, Shh. This molecule forms a gradient in the developing vertebrate neural tube which is interpreted by cells in the tissue through the actions of a Gene Regulatory Network (GRN) (for a recent review, see Ref. [37]). As a result of the concentration changing systematically along the neural tube, we are in fact dealing here with a family of networks that vary with neural tube position, parametrized below in terms of *p* running from 0 to 1 (where *p* also represents the Shh input). The network contains four molecular species (called transcription factors in the gene regulation context), two of which generate a bistable switch by mutual cross-repression as in our first example above. Similarly to how we proceeded in Ref. [27], we therefore place these two molecular species (Nkx2.2 and Olig2) in the subnetwork, with the bulk provided by the two other species (Irx3 and Pax6).

We test the method at the position along the neural tube where the model has the most complexity, a region of tristability (*p* = 0.65), and compare with the original system and the QSS approximation [Fig. 5(b)]. We find that as for the tetrastable example above (Fig. 2), the projection accurately replicates the choice of steady state, in contrast to the QSS method [Fig. 5(b)]. With the memory included, three basins of attraction are predicted, of which one—labeled pMN— separates the other two (p2 and p3) so no direct transitions from p2 to p3 can occur. The QSS approximation is not just quantitatively inaccurate but loses this biologically important qualitative feature, predicting instead that the p2 and p3 basins border each other. At other neural tube positions, we also consistently find a good match between the original system and the ZMs projection approach (not shown).

**FIG. 6:**
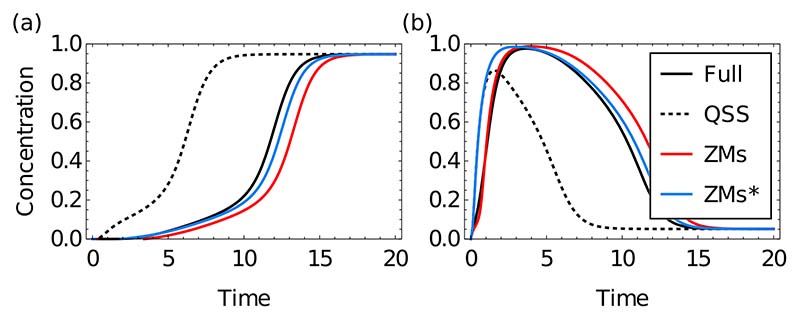
Time courses of concentrations of Nkx2.2 (a) and Olig2 (b) in p3 domain (*p* = 0.1). A transient expression of Olig2 leading to a delay in Nkx2.2 expression is observed *in vivo*. The full system (solid line) and ZMs projections [with Nkx2.2 and Olig2 chosen as subnetwork, see Fig. 5(a), red and blue] qualitatively reproduce this behavior. In contrast, the QSS approximation (dashed line) is unable to capture the long Olig2 transient. ZMs∗ represents the removal of all memory functions except those specified in Fig. 7(a), which suffice to capture the observed transient.

We next analyze the temporal evolution of the systems at various neural tube positions, using the experimentally determined initial condition for Nkx2.2 and Olig2 (vanishing concentration); we again compare the ZMs description with the original system and the QSS reduction. The ZMs predictions show a good fit with the original system at all positions (Fig. 6 displays results for a position with a strongly nonmonotonic transient, *p* = 0.1), demonstrating that the memory functions are capable of accurately capturing not just final cell fate decisions but also the timing of such decisions. This temporal aspect is important for correct patterning in the neural tube as explored in Ref. [38].

### A Decomposing nonlinear memory functions

**FIG. 7:**
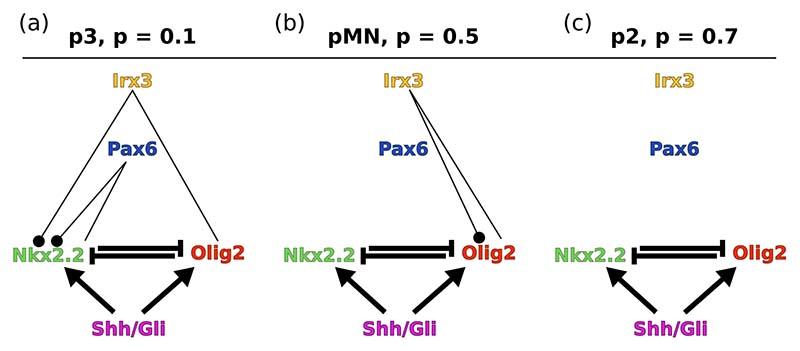
Diagrams indicating the channels that have the largest memory contributions to the observed dynamics at three distinct neural tube positions. Black dots indicate the species receiving the memory contribution; the other end of each line is the species “sending” the memory. Contributions change according to the final steady state: p3 (a), pMN (b) and p2 (c).

To understand how memory functions affect the patterning dynamics, we set out to understand their structure. We perform our self-consistent memory decomposition approach (Appendix E) and analyze the results to identify the memory channels with the most impact on the time courses based on their contribution along the trajectory (Appendix E). Performing this analysis for the different steady states, which along the neural tube form the so-called progenitor domains predicts the most important regulatory interactions contributing to the memory effect at each neural tube position (Fig. 7). This indicates marked differences in the most significant memory channels at different neural tube positions (see Appendix E).

To test the validity of our results we remove all channels identified as unimportant, setting them to QSS, thereby keeping only the channels shown in Fig. 7(a). Simulating the dynamics with only these memory functions results in dynamics that match closely those of the full simulation (Fig. 6), confirming the prediction that these channels dominate the memory effects.

We next investigated the experimentally validated transient in gene expression in the p3 domain (monostable with high Nkx2.2 in the steady state, *p* = 0.1, Fig. 6). Nkx2.2 induction is delayed in neural progenitor cells compared to Olig2 [39] and our analysis of the memory function provides insight into how this is achieved. The active memory channels ensure that Nkx2.2 is kept close to zero while Olig2 rises (Fig. 6). The dominant memory channels shown in Fig. 7(a) indicate that a different bulk species captures the history of each subnetwork species: Pax6 transmits the memory of Nkx2.2 and Irx3 the one of Olig2. The effect of these these bulk species is thus to delay Nkx2.2 expression based on the past expression of Nkx2.2 and Olig2.

**FIG. 8:**
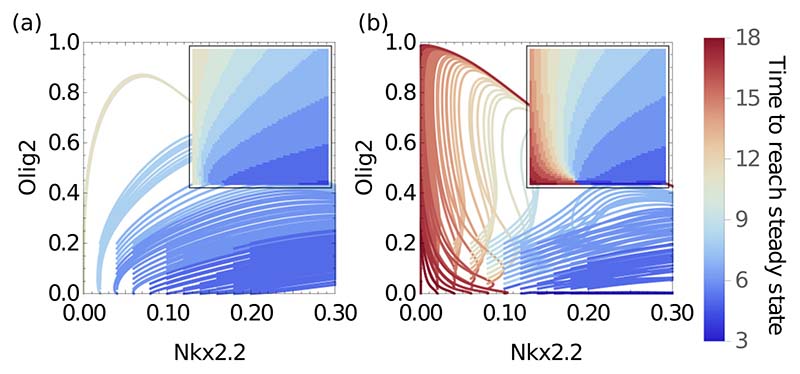
Trajectories from multiple initial conditions starting near zero for Olig2 and Nkx2.2 in the p3 region (*p* = 0.1). The comparison emphasises the robustness provided by the memory functions [ZMs, (b)] in comparison to a memoryless system [QSS, (a)]. All trajectories reach the same attractor with high Nkx2.2 and low Olig2. The ZMs system behaves almost identically to the full system, with transient increases in Olig2 and large delays in reaching the steady state from a variety of initial conditions. Color indicates time taken to reach final steady state, quantified as Nkx2.2 deviating less than 1% from its asymptotic concentration. Insets have same axes as main plots and show time to final steady state from a given initial position.

Finally, we examined whether the effect of the two memory functions (one for Olig2 and one for Nkx2.2) that reflect the influence of the bulk is to increase the robustness of the system to initial conditions. For the system with memory, the delay in Nkx2.2 expression is present for multiple initial conditions, with trajectories crossing in a way that would be impossible to reproduce with a memoryless system (Fig. 8). From Fig. 7(a), the memory has two dominant channels reacting to changes in Nkx2.2 and Olig2, respectively. This ensures that if even one of the subnetwork species levels drifts away from zero, the memory pushes the path back into the “correct” direction. In the case of the memoryless system, the already short transient observed in Fig. 6 disappears completely as soon as the initial conditions are no longer zero for Nkx2.2 [Fig. 8(a)]. In general, a transient is difficult to achieve in a 2D memoryless system where a very specific function would have to repress Nkx2.2 at both low and medium-high levels of Olig2. We find that the memory generated by the combination of Pax6 and Irx3 provides robustness to changes in the initial condition as the memory leads to low levels of Nkx2.2 during the initial phases of the transient [Fig. 8(b)].

## V Discussion

We have developed a version of the Zwanzig-Mori formalism, building on the work of Chorin *et al.* [11], to obtain closed-form memory functions that can be used to reduce the dimensionality of a dynamical system far from equilibrium. Our method applies to general first-order nonlinear differential equations of arbitrary dimensionality. Systems with higher order time derivatives can in principle also be treated by introducing auxiliary variables for the lower order time derivatives in the conventional way, though we have not explored examples of this type. The method is constrained only in that the bulk must have a unique steady state given the subnetwork state, which we refer to as QSS (quasi-steady state). This restriction can be viewed as analogous to the assumption in the standard Zwanzig-Mori projection that, due to detailed balance, the bulk always reaches a Gibbs distribution for a given subnetwork state.

Similar to the method of averaging and other dimensionality reduction approaches [5,17,40], our method contains a timescale separation assumption as its baseline, where the bulk is assumed to reach its QSS arbitrarily quickly, but in contrast it then systematically finds the memory terms that correct for this assumption.

In our examples, the bulk parts of the network were usually chosen as small because, given the nonlinear cross-repressive nature of the biological interactions that we study, multiple bulk steady states might otherwise result. However, there is no general restriction that the bulk has to be small *per se* or smaller than the subnetwork, as long as the assumption of a unique bulk steady state is met. For example, in the neural tube network, one could choose a single subnetwork species (Nkx2.2) and three bulk species.

We have demonstrated the accuracy of the approach in capturing emergent dynamics such as nontrivial transients and sustained or damped oscillations. By construction, the method can capture multistability and we have shown its accuracy in predicting the basins of attraction that, in a biological context, delineate cell fate decisions. We subsequently applied the method to a biologically relevant system, the neural tube patterning network [24]. The reduced model captures the nontrivial dynamics of the original system through its nonmonotonic transients. In addition, it provides an understanding of the cause of such transients in gene expression and suggests that memory effects, stored in independent bulk nodes, provide robustness to initial conditions.

As more information is acquired about complex systems, methods like ours that coarse grain the elements of a system but conserve the dynamics will be crucial to provide an understanding of such systems. We have demonstrated the generality of this method and its flexibility and applicability to dynamical systems. In the biological context, the approach holds promise, e.g., for more complex networks that incorporate signaling and gene regulation dynamics, where it could be applied to distinguish the impact of these two effects onto the macroscopic behavior in, e.g., tissue patterning.

In the present paper, we have not explored the role of the random force, which captures effects of the bulk initially being away from QSS. However, our method gives a closed-form approximation for this term in the projected equations, which is simply the function *F_s_* given by Eqs. (13) and (18). This should make it straightforward to explore random force effects in the future, e.g., to assess the importance of changes in bulk initial conditions in the spirit of our previous work [27].

A further interesting avenue of research would be the development of methods to capture the dynamics of systems even when parts of the network are unknown [19,20]. This approach could then be coupled with identifying the contribution of specific memory channels to the observed dynamics.

## Supplementary Material

Appendix
