# Supplementary material for: Tractable nonlinear memory functions as a tool to capture and explain dynamical behaviors
Source: Phys Rev Res. Author manuscript; Available in PMC 2023 Feb 27. (PMC7614247; doi:10.1103/PhysRevResearch.2.043069)
Supplement: Appendix [file EMS170583-supplement-Appendix.pdf]

APPENDIX A: EXPANSION AROUND QSS

In this Appendix, we detail how we derive the memory evolution over time. We make use of our

expansion Eq. (13) to find for the first term on the right hand side (RHS) of Eq. (5),

$$LF_s = \sum_b R_b(\boldsymbol{x}^{\rm s}, \boldsymbol{x}^{\rm b}) f_{bs}(\boldsymbol{x}^{\rm s}, \tau) + \sum_{s'} R_{s'}(\boldsymbol{x}^{\rm s}, \boldsymbol{x}^{\rm b}) \sum_b (x_b - x_b^*) \frac{\partial}{\partial x_{s'}} f_{bs}(\boldsymbol{x}^{\rm s}, \tau) - \sum_{s'} R_{s'}(\boldsymbol{x}^{\rm s}, \boldsymbol{x}^{\rm b}) \sum_b \frac{\partial x_b^*}{\partial x_{s'}} f_{bs}(\boldsymbol{x}^{\rm s}, \tau) \tag{A1}$$

where the two last terms arise by differentiating the product $(x_b - x_b^*) f_{bs}(\boldsymbol{x}^{\rm s}, \tau)$ w.r.t. $x_{s'}$ and we have not written the $\boldsymbol{x}^{\rm s}$ dependence of $\boldsymbol{x}^{\rm b*}$ for brevity. The second term on the RHS of Eq. (5) is the expectation of this, obtained by replacing $\boldsymbol{x}^{\rm b}$ by $\boldsymbol{x}^{\rm b*}$,

$$E[LF_s|\boldsymbol{x}^{\rm s}] = \sum_b R_b(\boldsymbol{x}^{\rm s}, \boldsymbol{x}^{\rm b*}) f_{bs}(\boldsymbol{x}^{\rm s}, \tau) - \sum_{s'} R_{s'}(\boldsymbol{x}^{\rm s}, \boldsymbol{x}^{\rm b*}) \sum_b \frac{\partial x_b^*}{\partial x_{s'}} f_{bs}(\boldsymbol{x}^{\rm s}, \tau). \tag{A2}$$

Putting the two together gives for the time evolution Eq. (5) of F_s:

$$\frac{\partial F_s}{\partial \tau} = \sum_b [R_b(\boldsymbol{x}^{\rm s}, \boldsymbol{x}^{\rm b}) - R_b(\boldsymbol{x}^{\rm s}, \boldsymbol{x}^{\rm b*})] f_{bs}(\boldsymbol{x}^{\rm s}, \tau) + \sum_{s'} R_{s'}(\boldsymbol{x}^{\rm s}, \boldsymbol{x}^{\rm b}) \sum_b (x_b - x_b^*) \frac{\partial}{\partial x_{s'}} f_{bs}(\boldsymbol{x}^{\rm s}, \tau)$$
$$- \sum_{s'b} [R_{s'}(\boldsymbol{x}^{\rm s}, \boldsymbol{x}^{\rm b}) - R_{s'}(\boldsymbol{x}^{\rm s}, \boldsymbol{x}^{\rm b*})] \frac{\partial x_b^*}{\partial x_{s'}} f_{bs}(\boldsymbol{x}^{\rm s}, \tau). \tag{A3}$$

For consistency with Eq. (13), we now linearize the square brackets again in $\boldsymbol{x}^{\rm b} - \boldsymbol{x}^{\rm b*}$. In the second term, we similarly replace $R_{s'}(\boldsymbol{x}^{\rm s}, \boldsymbol{x}^{\rm b})$ by $R_{s'}(\boldsymbol{x}^{\rm s}, \boldsymbol{x}^{\rm b*})$ as the remaining factor in this term is already linear. Comparing then with the time derivative of the original linearized formula Eq. (13) gives, after appropriate relabelling of indices, the equation for the evolution of f_{bs} in time Eq. (15).

From the above expansion, one sees that the matrix $l_{bb'}$ in Eq. (15) takes the form

$$l_{bb'} = \frac{\partial R_{b'}}{\partial x_b} - \sum_{s'} \frac{\partial R_{s'}}{\partial x_b} \frac{\partial x_{b'}^*}{\partial x_{s'}}. \tag{A4}$$

The form Eq. (16) in the main text is obtained by using the identity

$$\frac{\partial x_{b'}^*}{\partial x_{s'}} = -\sum_{b''} (\boldsymbol{J}^{-1})_{b'b''} \frac{\partial R_{b''}}{\partial x_{s'}}. \tag{A5}$$

The latter can be obtained by differentiating Eq. (9) with respect to x_s.

To obtain the actual memory function from Eq. (19) is now straightforward as we have already worked out the required expectation in Eq. (A2). The first term on the r.h.s. of Eq. (A2), which we had previously kept to make the ensuing linearization easier to see, actually vanishes because of Eq. (9), yielding

$$M_s(\boldsymbol{x}^{\rm s}, \tau) = -\sum_{s'b'} R_{s'} \frac{\partial x_{b'}^*}{\partial x_{s'}} f_{b's}(\boldsymbol{x}^{\rm s}, \tau). \tag{A6}$$

Using again the identity Eq. (A5), we obtain our main result Eq. (20).

APPENDIX B: SOLUTION FOR F

In this Appendix, we find the solution $f_{bs}(\boldsymbol{x}^{\rm s}, \tau)$ to the differential Eq. (15). We start by restating the latter as

$$\frac{\partial}{\partial \tau} f_{bs} - \sum_{s'} v_{s'}(\boldsymbol{x}^{\rm s}) \frac{\partial}{\partial x_{s'}} f_{bs} = \sum_{b'} l_{bb'}(\boldsymbol{x}^{\rm s}) f_{b's}, \tag{B1}$$

where we have used that the factor $R_{s'}$ in Eq. (15) is just the effective drift $v_{s'}$ defined in Eq. (11). As the equation is linear in f_{bs}, its derivatives it can be solved using the method of characteristics (see, e.g., Ref. [41]). Calling the curve parameter for a characteristic u, the characteristic equations can be read off from Eq. (B1) as

$$\frac{d\tau}{du} = 1, \tag{B2}$$

$$\frac{dx_s}{du} = -v_s(\boldsymbol{x}^{\rm s}), \tag{B3}$$

$$\frac{df_{bs}}{du} = \sum_{b'} l_{bb'}(\boldsymbol{x}^{\rm s}) f_{b's}. \tag{B4}$$

Setting an arbitrary integration constant to zero, the first of these gives $\tau = u$. To solve Eq. (B3), we call $\boldsymbol{\phi}_v$ the flow generated by $\boldsymbol{v}(\boldsymbol{x}^{\rm s})$, which is defined as the solution of the differential equations

$$\frac{\partial}{\partial \tau} \boldsymbol{\phi}_v(\boldsymbol{x}^{\rm s}, \tau) = \boldsymbol{v}(\boldsymbol{\phi}_v(\boldsymbol{x}^{\rm s}, \tau)), \qquad \boldsymbol{\phi}_v(\boldsymbol{x}^{\rm s}, 0) = \boldsymbol{x}^{\rm s}. \tag{B5}$$

The solution of Eq. (B3) is then

$$\boldsymbol{x}^{\rm s}(u) = \boldsymbol{\phi}_v(\boldsymbol{x}_0^{\rm s}, -u), \tag{B6}$$

where $\boldsymbol{x}_0^{\rm s}$ is the value at the beginning of the characteristic curve ($u = 0$); the minus sign in the second argument of $\boldsymbol{\phi}_v$ reflects the backward in time propagation in Eq. (B3). We note for later that, as a consequence of Eq. (B6), the solution values at u_1 and u_2 are related by

$$\boldsymbol{x}^{\rm s}(u_2) = \boldsymbol{\phi}_v(\boldsymbol{x}^{\rm s}(u_1), -u_2 + u_1). \tag{B7}$$

Finally, the solution of Eq. (B4) is

$$f_{bs}(u) = \sum_{b'} \left(e^{\int_0^u du' \boldsymbol{l}(\boldsymbol{x}^{\rm s}(u'))}\right)_{bb'} f_{b's}^0(\boldsymbol{x}_0^{\rm s}), \tag{B8}$$

using the initial condition Eq. (14) at $\tau = u = 0$. From Eq. (B4), we see that the matrix exponential appearing here must be time ordered, with earlier "times" u' appearing to the right of later ones.

It now remains to express $f_{bs}(u)$ in terms of $\boldsymbol{x}^{\rm s}(u)$ and $\tau(u) = u$. We fix a $\hat{u} = \hat{\tau}$ and call $\hat{\boldsymbol{x}}^{\rm s} = \boldsymbol{x}^{\rm s}(\hat{u})$. Using Eq. (B7)

with $u_2 = u'$ and $u_1 = u$ then shows that the $\boldsymbol{x}^{\rm s}$-solution Eq. (B6) can be expressed in terms of $\hat{\boldsymbol{x}}^{\rm s}$ as

$$\boldsymbol{x}^{\rm s}(u') = \boldsymbol{\phi}_v(\hat{\boldsymbol{x}}^{\rm s}, \hat{\tau} - u') \tag{B9}$$

and, in particular, $\boldsymbol{x}_0^{\rm s} = \boldsymbol{\phi}_v(\hat{\boldsymbol{x}}^{\rm s}, \hat{\tau})$, so

$$f_{bs}(\hat{\boldsymbol{x}}^{\rm s}, \hat{\tau}) = \sum_{b'} \left(e^{\int_0^{\hat{\tau}} du'\, \boldsymbol{l}(\boldsymbol{\phi}_v(\hat{\boldsymbol{x}}^{\rm s}, \hat{\tau}-u'))}\right)_{bb'} f^0_{b's}(\boldsymbol{\phi}_v(\hat{\boldsymbol{x}}^{\rm s}, \hat{\tau})). \tag{B10}$$

Changing the integration variable to $\tau' = \hat{\tau} - u'$ and dropping the hats then gives the solution Eq. (18) announced in the main text. Note that as $\tau' = \hat{\tau} - u'$, the time ordering of the matrix exponential,

$$\boldsymbol{E}(\tau) = \exp\left(\int_0^\tau d\tau'\, \boldsymbol{l}(\boldsymbol{\phi}_v(\boldsymbol{x}^{\rm s}, \tau'))\right), \tag{B11}$$

is such that the earlier τ' are now on the *left*. The appropriate time-ordered matrix exponential is defined formally via its Taylor series,

$$\boldsymbol{E}(\tau) = \boldsymbol{1} + \sum_{n=1}^{\infty} \int \prod_{i=1}^{n} d\tau_i\, \boldsymbol{l}(\boldsymbol{\phi}_v(\boldsymbol{x}^{\rm s}, \tau_1)) \times \cdots \times \boldsymbol{l}(\boldsymbol{\phi}_v(\boldsymbol{x}^{\rm s}, \tau_n)), \tag{B12}$$

with $\boldsymbol{1}$ the identity matrix and the integration in the other terms running over the range $0 < \tau_1 < \ldots < \tau_n < \tau$.

APPENDIX C: MAPPING OF SELF-CONSISTENT MEMORY TO DIFFERENTIAL EQUATIONS

We show in this Appendix how to map the subnetwork equations with self-consistent memory,

$$\frac{\partial}{\partial t} x_s = v_s(\boldsymbol{x}^{\rm s}(t)) + \tilde{\mathcal{M}}_s(t), \tag{C1}$$

to a set of differential equations. The self-consistent memory term $\tilde{\mathcal{M}}_s(t)$ is given by Eq. (24),

$$\tilde{\mathcal{M}}_s(t) = \sum_{b''} \int_0^t dt' \sum_{b'} c_{b'}(\boldsymbol{x}^{\rm s}(t'))\left(e^{\int_{t'}^t dt''\, \boldsymbol{l}(\boldsymbol{x}^{\rm s}(t''))}\right)_{b'b''} f^0_{b''s}(\boldsymbol{x}^{\rm s}(t)), \tag{C2}$$

so can be written as

$$\tilde{\mathcal{M}}_s(t) = \sum_b m_b(t) f^0_{bs}(\boldsymbol{x}^{\rm s}(t)), \tag{C3}$$

with

$$m_b(t) = \int_0^t dt' \sum_{b'} c_{b'}(\boldsymbol{x}^{\rm s}(t'))\left(e^{\int_{t'}^t dt''\, \boldsymbol{l}(\boldsymbol{x}^{\rm s}(t''))}\right)_{b'b}. \tag{C4}$$

It is then straightforward to check that

$$\frac{d}{dt} m_b(t) = c_b(\boldsymbol{x}^{\rm s}(t)) + \sum_{b'} m_{b'}(t) l_{b'b}(\boldsymbol{x}^{\rm s}(t)), \tag{C5}$$

where the second term arises from the t dependence of the matrix exponential. The $m_b(t)$ can therefore be obtained numerically by integrating the differential Eq. (C5) together with the subnetwork equations with (self-consistent) memory:

$$\frac{d}{dt} x_s(t) = v_s(\boldsymbol{x}^{\rm s}(t)) + \sum_{b'} m_{b'}(t) f^0_{sb'}(\boldsymbol{x}^{\rm s}(t)). \tag{C6}$$

The appropriate initial conditions for the auxiliary variables follow from Eq. (C4) as $m_b(0) = 0$.

APPENDIX D: CHANNEL DECOMPOSITION

We begin by writing the expression for the memory function explicitly, combining Eqs. (14), (18), (20), and (21):

$$M_s(\boldsymbol{x}^{\rm s}, \tau) = \sum_{b's'b''} (\boldsymbol{J}^{-1})_{b'b''} \frac{\partial R_{b''}}{\partial x_{s'}} R_{s'} f_{b's}(\boldsymbol{x}^{\rm s}, \tau), \tag{D1}$$

$$= \sum_{b's'b''} (\boldsymbol{J}^{-1})_{b'b''} \frac{\partial R_{b''}}{\partial x_{s'}} R_{s'} \sum_c E_{b'c}(\tau) f^0_{cs}(\boldsymbol{\phi}_v(\boldsymbol{x}^{\rm s}, \tau)), \tag{D2}$$

$$= \sum_{b's'b''} (\boldsymbol{J}^{-1})_{b'b''} \frac{\partial R_{b''}}{\partial x_{s'}} R_{s'} \sum_c E_{b'c}(\tau) \frac{\partial R_s}{\partial x_c}(\boldsymbol{\phi}_v(\boldsymbol{x}^{\rm s}, \tau)), \tag{D3}$$

where the first three factors are evaluated at $\boldsymbol{x}^{\rm s}$. We now swap index labels and group the sums into a more intuitive form:

$$M_s(\boldsymbol{x}^{\rm s}, \tau) = \sum_{s'} \sum_{bb'} \left(\sum_{b''} (\boldsymbol{J}^{-1})_{b''b'}(\boldsymbol{x}^{\rm s}) \frac{\partial R_{b'}}{\partial x_{s'}}(\boldsymbol{x}^{\rm s}) R_{s'}(\boldsymbol{x}^{\rm s}) E_{b''b}(\tau) \frac{\partial R_s}{\partial x_b}(\boldsymbol{\phi}_v(\boldsymbol{x}^{\rm s}, \tau)) \right). \tag{D4}$$

As discussed in the main text, the expression up to before the exponential represents a change in the deviation of the bulk species concentration $x_{b''}$ from its QSS values over some small time interval, in response to changes in the subnetwork concentrations $x_{s'}$ [see also Eq. (I4) below]. In the factor $\partial R_{b'}/\partial x_{s'}$, only those bulk species b' contribute whose time evolution depends explicitly on the subnetwork species s' driving the bulk time evolution via $R_{s'}$. The b' can then be interpreted as *outgoing channels* for the signal from s'. After propagation in the bulk network, the signal returns via another bulk species. Here only bulk species b contribute that appear explicitly in the time evolution of subnetwork species s as indicated by the factors $\partial R_s/\partial x_b$. The b can therefore be interpreted as *incoming channels*. Overall, we have memory effects from s' onto s, via an outgoing channel (s' to b') and an incoming channel (b to s). Consistent with this interpretation, the outgoing channel "susceptibilities" $\partial R_{b'}/\partial x_{s'}$ are evaluated for the past, i.e., sending, state $\boldsymbol{x}^{\rm s} \equiv \boldsymbol{x}^{\rm s}(t')$ of the subnetwork. The incoming channel susceptibilities $\partial R_s/\partial x_b$, on the other hand, are evaluated at the current time t as shown by the propagation via $\boldsymbol{\phi}_v$ across the time difference $\tau = t - t'$. Within the self-consistent approximation Eq. (C5), this

propagation corresponds directly to evaluation at the current state $\boldsymbol{x}^{\mathrm{s}}(t)$.

APPENDIX E: SELF-CONSISTENT CHANNEL DECOMPOSITION

The channel decomposition of Sec. D can also be applied to the self-consistent memory approximation, as we now outline. Writing out the self-consistent memory term Eq. (C2) explicitly and reordering and relabelling terms as in Eq. (D4) gives

$$\begin{aligned}\tilde{\mathcal{M}}_s(t) &= \int_0^t dt' \sum_{s'}\sum_{bb'}\sum_{b''} \frac{\partial R_s}{\partial x_b}(\boldsymbol{x}^{\mathrm{s}}(t))\left(e^{\int_{t'}^{t} dt'' \boldsymbol{l}(\boldsymbol{x}^{\mathrm{s}}(t''))}\right)_{b''b} \\ &\quad\times (\boldsymbol{J}^{-1})_{b''b'}(\boldsymbol{x}^{\mathrm{s}}(t'))\frac{\partial R_{b'}}{\partial x_{s'}}(\boldsymbol{x}^{\mathrm{s}}(t'))R_{s'}(\boldsymbol{x}^{\mathrm{s}}(t')) \\ &= \sum_{s'}\sum_{bb'} \frac{\partial R_s}{\partial x_b}(\boldsymbol{x}^{\mathrm{s}}(t)) m_{sbb's'}(t),\end{aligned} \tag{E1}$$

where

$$\begin{aligned} m_{sbb's'}(t) &= \int_0^t dt' \sum_{b''}(\boldsymbol{J}^{-1})_{b''b'}(\boldsymbol{x}^{\mathrm{s}}(t'))\frac{\partial R_{b'}}{\partial x_{s'}}(\boldsymbol{x}^{\mathrm{s}}(t'))R_{s'}(\boldsymbol{x}^{\mathrm{s}}(t')) \\ &\quad\times \left(e^{\int_{t'}^{t} dt'' \boldsymbol{l}(\boldsymbol{x}^{\mathrm{s}}(t''))}\right)_{b''b}.\end{aligned} \tag{E2}$$

From this last representation, it follows that the $m_{sbb's'}(t)$ vanish at $t = 0$ and obey the differential equations:

$$\begin{aligned}\frac{d}{dt} m_{sbb's'}(t) &= (\boldsymbol{J}^{-1})_{bb'}(\boldsymbol{x}^{\mathrm{s}}(t))\frac{\partial R_{b'}}{\partial x_{s'}}(\boldsymbol{x}^{\mathrm{s}}(t))R_{s'}(\boldsymbol{x}^{\mathrm{s}}(t)) \\ &\quad+ \sum_{b''} m_{sb''b's'}(t) l_{b''b}(\boldsymbol{x}^{\mathrm{s}}(t)).\end{aligned} \tag{E3}$$

The channel-decomposed memory can therefore also be calculated from differential equations (see Appendix G). Of course, one only needs to find the $m_{sbb's'}$ for combinations (sb) and $(b's')$, where the corresponding channel susceptibilities are nonzero.

APPENDIX F: EXACTNESS OF MEMORY

We show that the self-consistent memory $m_b(t)$ is exact when both R_s and R_b contain at most linear terms in $\boldsymbol{x}^{\mathrm{b}}$. In such a case, the full system can be written as

$$R_s = v_s + \sum_{b'} \tilde{x}_{b'} f^0_{b's}, \qquad R_b = \sum_{b'} J_{bb'}\tilde{x}_{b'}, \tag{F1}$$

where $\tilde{x}_b = x_b - x^*_b(\boldsymbol{x}^{\mathrm{s}})$ and the QSS value $\boldsymbol{x}^{\mathrm{b}*}(\boldsymbol{x}^{\mathrm{s}})$ is an arbitrary function of $\boldsymbol{x}^{\mathrm{s}}$. We now want to show that the $\tilde{x}_b$ correspond *exactly* to the m_b from the self-consistent ZMs method. To do this, we work out their evolution in time:

$$\begin{aligned}\frac{d}{dt}\tilde{x}_b &= R_b - \sum_{s'} R_{s'}\frac{\partial x^*_b}{\partial x_{s'}} \\ &= \sum_{b'} J_{bb'}\tilde{x}_{b'} + \sum_{s'}\left(v_{s'} + \sum_{b'}\tilde{x}_{b'} f^0_{b's'}\right)\sum_{b''}(\boldsymbol{J}^{-1})_{bb''}\frac{\partial R_{b''}}{\partial x_{s'}}.\end{aligned} \tag{F2}$$

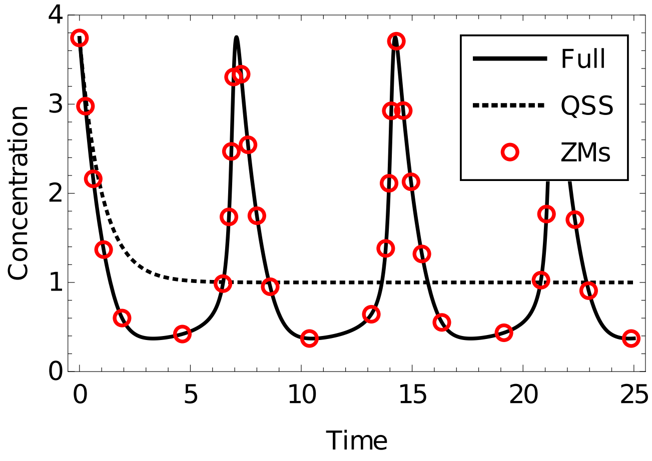

FIG. 9. Oscillating Brusselator system as described in Ref. [31]; we retain the concentration of the first species x_1 in the subnetwork and place the second species in the bulk. Parameters for the sustained oscillatory regime in this example are $A = 1$ and $B = 3$ in the notation of Ref. [31]. The trajectory of the self-consistent projection (red dots) captures that of the original system (solid line) exactly as expected from the general proof in Appendix F, while the QSS approximation (dotted line) fails qualitatively.

By using that for an $\boldsymbol{x}^{\mathrm{b}}$-linear system as assumed here, one has $f^0_{bs} = \partial R_s/\partial x_b$, the above can be rewritten as

$$\begin{aligned}\frac{d}{dt}\tilde{x}_b &= \sum_{b''}(\boldsymbol{J}^{-1})_{bb''}\frac{\partial R_{b''}}{\partial x_{s'}}v_{s'} \\ &\quad+ \sum_{b'}\tilde{x}_{b'}\left(J_{bb'} + \sum_{s'b''}(\boldsymbol{J}^{-1})_{bb''}\frac{\partial R_{b''}}{\partial x_{s'}}\frac{\partial R_{s'}}{\partial x_{b'}}\right).\end{aligned} \tag{F3}$$

Using then the definitions Eqs. (16) and (21), we obtain an expression equivalent to Eq. (C5),

$$\frac{d}{dt}\tilde{x}_b = c_b + \sum_{b'}\tilde{x}_{b'} l_{b'b}, \tag{F4}$$

thus showing that $\tilde{x}_b = m_b$ when we start from the same initial condition $\tilde{x}_b = 0$, i.e., the bulk at QSS.

We test the above exactness statement on two different examples that have a linear dependence on a particular species but nonlinear dependencies on other species: a minimal bistable system as described in Ref. [30], and the Brusselator, which is capable of achieving limit cycles [31]. As expected from the above derivation, the self-consistent memory captures the behavior of both systems *exactly* (Figs. 9 and 10). As further shown in Fig 10, the original nonlinear projection method ZMn is also accurate at capturing the dynamics though not necessarily exact. The corresponding memory function is shown in Fig.11. (We note that the memory functions of the Brusselator grow exponentially in a way that forces memory terms to cancel out to zero at the fixed point; this leads to numerical challenges that we do not pursue here.)

APPENDIX G: LINEAR DYNAMICS

We discuss briefly the case of fully linear dynamics, where the dependence of R_s and R_b on *all* variables $\boldsymbol{x}^{\mathrm{s}}$ and $\boldsymbol{x}^{\mathrm{b}}$ (not

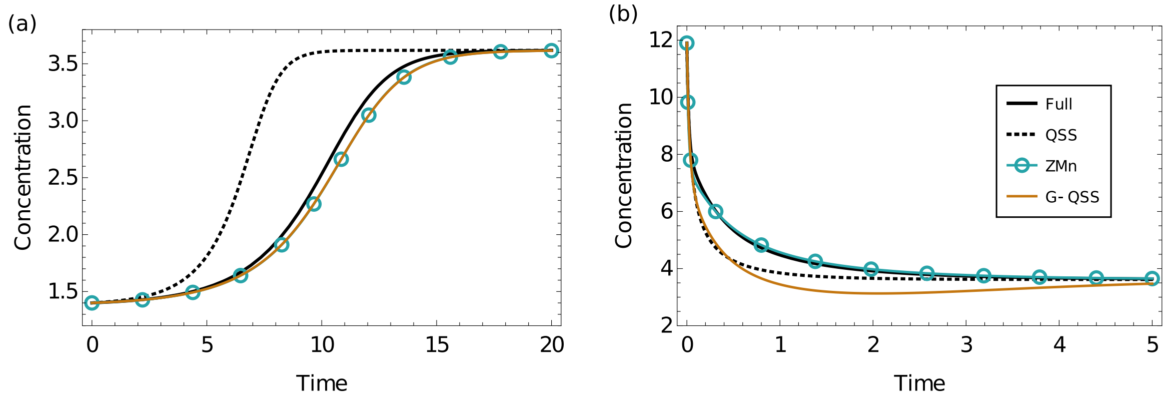

FIG. 10. Minimal bistable system with linear dependence on the second species as described in Ref. [30]; we choose x_1 for the subnetwork and place the second species in the bulk. Parameters for the bistable regime in this example are $k_1 = 10$, $k_2 = 1$, $k_3 = 2$, and $k_4 = 1$ [30]. The trajectory of the self-consistent ZMs projection again captures that of the original system (solid line) exactly (see Appendix F and Fig. 9) so is not plotted. G-QSS represents the Gouasmi *et al.* approach adapted to QSS projection (Appendix I). (a) $x_1(0) = 1.4$. At initial conditions near the fixed point, both the ZMn method (cyan dots) and G-QSS (orange line) behave similarly and accurately capture the full dynamics. (b) $x_1(0) = 11.9$. Further away from the final stable fixed point, the G-QSS predictions become increasingly inaccurate while the ZMn method continues to provide a good approximation.

just $\boldsymbol{x}^{\mathrm{b}}$ as in Appendix F) is only via constant and linear terms. Such a description can always be obtained by expanding linearly around a fixed point of the dynamics [21,27]. One then sees from Eqs. (16) and (14) that $l_{bb'}$ and f^0_{bs} are both constant, i.e., independent of $\boldsymbol{x}^{\mathrm{s}}$. Accordingly [compare Eqs. (18), (20), and (24)]. the memory functions of the ZMn and ZMs projections also become identical, and the corresponding channel decompositions are also the same.

To illustrate the linearized dynamics approach, we perform a channel decomposition of the amplitude (value at $\tau = 0$) of the linearized memory in the neural tube system as we did in Ref. [27], but now for the method derived in this study (Fig. 12). We find similar profiles to those found in Ref. [27]. The results highlight the relative weakness of the memory from Olig2 into Nkx2.2 via Pax6, supporting the conclusions of Ref. [27]. In addition, channel decomposition for the ZM trajectory shown in Fig. 6 provided memory terms affecting Nkx2.2 and Olig2 (Fig. 13). The method derived in this study is, however, significantly more powerful as it does not rely on an expansion near a steady state and gives access to the full memory and its channel decomposition as described in Appendixes D and E.

APPENDIX H: COMPARISON WITH ALTERNATIVE MEMORY FUNCTION APPROXIMATION

Gouasmi *et al.* [18] propose an approximation for the memory function for the case where the projection Eq. (10) is defined not by setting the bulk coordinates to their $\boldsymbol{x}^{\mathrm{s}}$-dependent QSS values, but simply to zero:

$$E[g(\cdot)|\boldsymbol{x}^{\mathrm{s}}] = g(\boldsymbol{x}^{\mathrm{s}}, 0). \tag{H1}$$

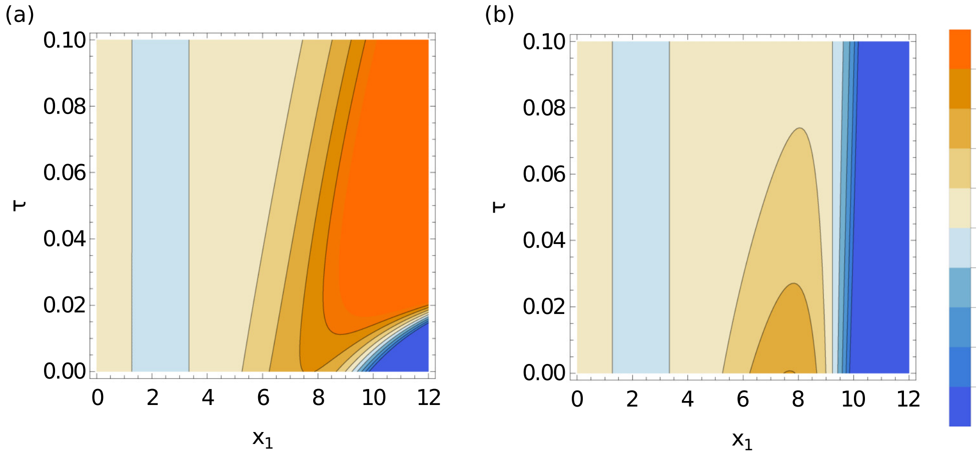

FIG. 11. Memory functions for the system detailed in Ref. [30] with the parameters chosen for Fig. 10. (a) Using the ZMn. (b) Using the method from Gouasmi *et al.* as extended to QSS projection in Appendix I. The x axis shows the concentration of the subnetwork species x_1 while the y axis indicates time difference τ. By construction, the two memory function approximations predict the same value (scale bar to the right) at $\tau = 0$, as they only differ in how they propagate the memory over time. At $\tau > 0$, the memory functions are relatively similar for $x_1 \in [0, 6]$ but become progressively different as x_1 grows beyond this range; for $x_1 \geqslant 10$, the G-QSS method predicts a negative memory function for all τ that leads to its poor performance as observed in Fig. 10.

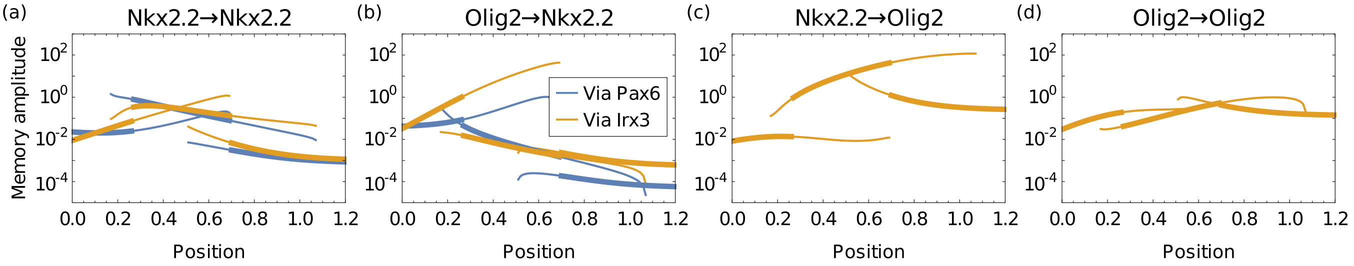

FIG. 12. Amplitudes (value at time difference $\tau = 0$) of nonlinear memory functions linearized around fixed points, for comparison with the approach of Ref. [27], where memory functions expanded around fixed points were calculated directly. (a) Amplitude of memory of Nkx2.2 to itself along the neural tube. There are multiple lines as the analysis was performed at all possible stable steady states. The vertical axis is logarithmic to make the range of amplitudes easier to appreciate. Colors identify the memory amplitude contribution from the two possible bulk channels, via Irx3 and Pax6, respectively. Thick lines indicate physiological states, while thin lines indicate states that are not usually observed in vivo. (b) Amplitude of memory of Nkx2.2 to levels of Olig2, shown along the neural tube. The memory via Pax6 is for the most part below the memory via Irx3, in each pair of corresponding curves. (c), (d) Amplitudes of memory of Olig2 to past Nkx2.2 (c) and to itself (d). No channel decomposition is performed as Olig2 receives memory only via the Irx3 channel.

The function $F_s(\boldsymbol{x}^{\mathrm{s}}, \boldsymbol{x}^{\mathrm{b}}, \tau)$ still evolves according to Eq. (5), which written out now reads

$$\frac{\partial}{\partial\tau}F_s = LF_s(\boldsymbol{x}^{\mathrm{s}}, \boldsymbol{x}^{\mathrm{b}}, \tau) - E[LF_s(\cdot, \tau)|\boldsymbol{x}^{\mathrm{s}}], \tag{H2}$$

$$= \sum_{s'} R_{s'}(\boldsymbol{x}^{\mathrm{s}}, \boldsymbol{x}^{\mathrm{b}})\frac{\partial F_s}{\partial x_{s'}} + \sum_b R_b(\boldsymbol{x}^{\mathrm{s}}, \boldsymbol{x}^{\mathrm{b}})\frac{\partial F_s}{\partial x_b},$$

$$- \sum_{s'} R_{s'}(\boldsymbol{x}^{\mathrm{s}}, 0)\frac{\partial F_s}{\partial x_{s'}}(\boldsymbol{x}^{\mathrm{s}}, 0, \tau)$$

$$- \sum_b R_b(\boldsymbol{x}^{\mathrm{s}}, 0)\frac{\partial F_s}{\partial x_b}(\boldsymbol{x}^{\mathrm{s}}, 0, \tau), \tag{H3}$$

where the very last factor is the x_b derivative of F evaluated at $\boldsymbol{x}^{\mathrm{b}} = 0$. The approximation of Ref. [18] amounts to ignoring the fact that the derivatives of F_s are evaluated at a different point in the last two lines, which gives

$$\frac{\partial}{\partial\tau}F_s = \sum_{s'}[R_{s'}(\boldsymbol{x}^{\mathrm{s}}, \boldsymbol{x}^{\mathrm{b}}) - R_{s'}(\boldsymbol{x}^{\mathrm{s}}, 0)]\frac{\partial F_s}{\partial x_s}$$

$$+ \sum_b [R_b(\boldsymbol{x}^{\mathrm{s}}, \boldsymbol{x}^{\mathrm{b}}) - R_b(\boldsymbol{x}^{\mathrm{s}}, 0)]\frac{\partial F_s}{\partial x_b}. \tag{H4}$$

This has the form of a Liouville equation as noticed in Ref. [18] and so its solution can be written as

$$F_s(\boldsymbol{x}^{\mathrm{s}}, \boldsymbol{x}^{\mathrm{b}}, \tau) = F_s(\boldsymbol{\psi}^{\mathrm{s}}(\boldsymbol{x}^{\mathrm{s}}, \boldsymbol{x}^{\mathrm{b}}, \tau), \boldsymbol{\psi}^{\mathrm{b}}(\boldsymbol{x}^{\mathrm{s}}, \boldsymbol{x}^{\mathrm{b}}, \tau)), \tag{H5}$$

where the components of the vector functions $\boldsymbol{\psi}^{\mathrm{s}}$ and $\boldsymbol{\psi}^{\mathrm{b}}$ evolve with τ according to

$$\frac{\partial}{\partial\tau}\psi_s = R_s(\boldsymbol{\psi}^{\mathrm{s}}, \boldsymbol{\psi}^{\mathrm{b}}) - R_s(\boldsymbol{\psi}^{\mathrm{s}}, 0), \tag{H6}$$

$$\frac{\partial}{\partial\tau}\psi_b = R_b(\boldsymbol{\psi}^{\mathrm{s}}, \boldsymbol{\psi}^{\mathrm{b}}) - R_b(\boldsymbol{\psi}^{\mathrm{s}}, 0), \tag{H7}$$

from the initial conditions

$$\psi_b(\boldsymbol{x}^{\mathrm{s}}, \boldsymbol{x}^{\mathrm{b}}, 0) = x_b, \qquad \psi_s(\boldsymbol{x}^{\mathrm{s}}, \boldsymbol{x}^{\mathrm{b}}, 0) = x_s. \tag{H8}$$

The function F_s at $\tau = 0$, which as before we write without a time argument, is given by the analog of Eq. (12):

$$F_s(\boldsymbol{x}^{\mathrm{s}}, \boldsymbol{x}^{\mathrm{b}}) = R_s(\boldsymbol{x}^{\mathrm{s}}, \boldsymbol{x}^{\mathrm{b}}) - R_s(\boldsymbol{x}^{\mathrm{s}}, 0). \tag{H9}$$

The corresponding memory as defined in Eq. (19) is

$$M_s^{\mathrm{G}}(\boldsymbol{x}^{\mathrm{s}}, \tau) = \sum_{s'} R_{s'}\frac{\partial}{\partial x_{s'}}F_s(\boldsymbol{\psi}^{\mathrm{s}}, \boldsymbol{\psi}^{\mathrm{b}}) + \sum_b R_b\frac{\partial}{\partial x_b}F_s(\boldsymbol{\psi}^{\mathrm{s}}, \boldsymbol{\psi}^{\mathrm{b}}), \tag{H10}$$

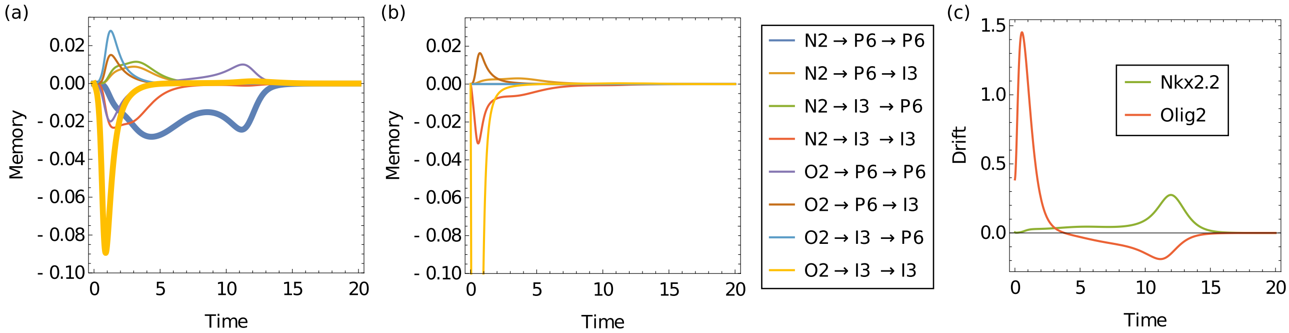

FIG. 13. Channel decomposition of the memory terms for the ZMs trajectory shown in Fig. 6. (a) and (b) show memory terms affecting Nkx2.2 and Olig2, respectively. Each color represents a memory channel as indicated. The memory originates from a particular subnetwork species "sending" memory through a specific bulk species; the effect then propagates within the bulk and returns via a specific bulk species (see legend). The two most salient memory functions are Nkx2.2 to Pax6 and then returning through Pax6 into Nkx2.2 (thick yellow line), and Olig2 to Irx3 and then returning through Pax6 into Nkx2.2 (thick blue line). (b) shows a large memory contribution acting on Olig2 via the channel through Irx3 (yellow line). However, in this case, the drift for Olig2 (c) is so large that the relative effect of this memory channel remains nonetheless small.

where all $R_{s'}$, R_b and the derivatives are evaluated at $(\boldsymbol{x}^{\rm s}, 0)$. Gouasmi *et al.* propose to find these derivatives numerically, but in fact a closed form expression can be obtained as follows. Applying the chain rule gives

$$M_s^{\rm G}(\boldsymbol{x}^{\rm s},\tau)=\sum_{s's''}R_{s'}\frac{\partial F_s}{\partial\psi_{s''}}\frac{\partial\psi_{s''}}{\partial x_{s'}}+\sum_{s'b}R_{s'}\frac{\partial F_s}{\partial\psi_b}\frac{\partial\psi_b}{\partial x_{s'}}+\sum_{s'b}R_b\frac{\partial F_s}{\partial\psi_{s'}}\frac{\partial\psi_{s'}}{\partial x_b}+\sum_{bb'}R_b\frac{\partial F_s}{\partial\psi_{b'}}\frac{\partial\psi_{b'}}{\partial x_b}. \tag{H11}$$

Now note that in the final evaluation we always use $\boldsymbol{x}^{\rm b}=0$, which from the differential Eqs. (H6) and (H7) implies $\boldsymbol{\psi}^{\rm s}=\boldsymbol{x}^{\rm s}$, $\boldsymbol{\psi}^{\rm b}=0$ for all τ. Hence, in particular ψ_b is independent of $\boldsymbol{x}^{\rm s}$ and so $\partial\psi_b/\partial x_{s'}=0$. We also have $F_s(\boldsymbol{x}^{\rm s},0)=0$ from Eq. (H9), which implies $\partial F_s/\partial\psi_{s'}=0$. Only the last term from Eq. (H11) thus survives,

$$M^{\rm G}(\boldsymbol{x}^{\rm s},\tau)=\sum_{bb'}R_b\frac{\partial F_s}{\partial\psi_{b'}}\frac{\partial\psi_{b'}}{\partial x_b}, \tag{H12}$$

and it remains to find $\partial\psi_{b'}/\partial x_b$. By differentiating Eq. (H7) for $\partial\psi_{b'}/\partial\tau$ w.r.t. x_b, one finds

$$\frac{\partial}{\partial\tau}\frac{\partial\psi_{b'}}{\partial x_b}=\frac{\partial R_{b'}}{\partial\psi_{b''}}\frac{\partial\psi_{b''}}{\partial x_b}. \tag{H13}$$

On the r.h.s., a similar term from the variation of $\boldsymbol{\psi}^{\rm s}$ vanishes because it would be proportional to

$$\frac{\partial R_{b'}}{\partial\psi_s}(\boldsymbol{\psi}^{\rm s},\boldsymbol{\psi}^{\rm b})-\frac{\partial R_{b'}}{\partial\psi_s}(\boldsymbol{\psi}^{\rm s},0). \tag{H14}$$

This difference is zero in the final evaluation at $\boldsymbol{x}^{\rm b}=0$ (which implies $\boldsymbol{\psi}^{\rm b}=0$). For the same reason, the derivatives $\partial R_{b'}/\partial\psi_{b''}=\partial R_{b'}/\partial x_{b''}$ are evaluated at $(\boldsymbol{x}^{\rm s},0)$ and constant in time τ. Collecting these derivatives into a matrix $\boldsymbol{k}$ with elements $k_{b'b''}$ and using that $\partial\psi'_b/\partial x_b=\delta_{bb'}$ ($=1$ for $b=b'$ and $=0$ otherwise) at $\tau=0$ gives then as the explicit solution of Eq. (H13),

$$\frac{\partial\psi_{b'}}{\partial x_b}=(e^{\boldsymbol{k}\tau})_{b'b}, \tag{H15}$$

and inserting into Eq. (H12) yields

$$M^{\rm G}(\boldsymbol{x}^{\rm s},\tau)=\sum_{bb'}\frac{\partial R_s}{\partial x_{b'}}(e^{\boldsymbol{k}\tau})_{b'b}R_b, \tag{H16}$$

where we have used that $\partial F_s/\partial\psi_{b'}=\partial F_s/\partial x_{b'}=\partial R_s/\partial x_{b'}$; this derivative and the factor R_b are evaluated at $(\boldsymbol{x}^{\rm s},0)$ in the approximation from Ref. [18] for the memory function.

We do not show here how the above memory approximation performs in our test systems because the nature of the approach can lead to fixed points disappearing after projection or new fixed points appearing. We observed both of these effects in numerical evaluations for the bistable switch from Ref. [30].

APPENDIX I: EXTENDING GOUASMI *ET AL.* APPROXIMATION WITH QSS PROJECTION

The Gouasmi *et al.* approximation [18] for the memory function rests on projecting to $\boldsymbol{x}^{\rm b}=0$, but this is not generally an appropriate baseline for our case as it would correspond to setting all bulk concentrations to zero. However, we can adapt the approximation to the spirit of our work by changing coordinate system so that zero bulk coordinates correspond to the projection we consider throughout the paper, i.e., to QSS bulk concentrations. Explicitly, this variable transformation reads

$$\tilde{x}_s=x_s,\qquad \tilde{x}_b=x_b-x_b^*(\boldsymbol{x}^{\rm s}) \tag{I1}$$

because $\tilde{x}_b=0$ is then equivalent to $x_b=x_b^*(\boldsymbol{x}^{\rm s})$. The time evolution of the new variables follows as

$$\frac{d}{dt}\tilde{x}_s=\tilde{R}_s(\tilde{\boldsymbol{x}}^{\rm s},\tilde{\boldsymbol{x}}^{\rm b})=R_s(\tilde{\boldsymbol{x}}^{\rm s},\boldsymbol{x}^{\rm b*}+\tilde{\boldsymbol{x}}^{\rm b}), \tag{I2}$$

$$\frac{d}{dt}\tilde{x}_b=\tilde{R}_b(\tilde{\boldsymbol{x}}^{\rm s},\tilde{\boldsymbol{x}}^{\rm b}), \tag{I3}$$

$$=R_b(\tilde{\boldsymbol{x}}^{\rm s},\boldsymbol{x}^{\rm b*}+\tilde{\boldsymbol{x}}^{\rm b})+\sum_s\left[\sum_{b'}(\boldsymbol{J}^{-1})_{bb'}\frac{\partial R_{b'}}{\partial x_s}\right]\times R_s(\tilde{\boldsymbol{x}}^{\rm s},\boldsymbol{x}^{\rm b*}+\tilde{\boldsymbol{x}}^{\rm b}), \tag{I4}$$

where the factors enclosed in square brackets are the explicit expression for $-\partial x_b^*/\partial x_s$ and have to be evaluated at $\boldsymbol{x}^{\rm b*}$.

The Gouasmi memory function approximation, adapted for our QSS projection, is now given by Eq. (H16) applied to the new variables $\tilde{x}_s$, $\tilde{x}_b$ and corresponding drift functions $\tilde{R}_s$, $\tilde{R}_b$. The last factor is $\tilde{R}_b(\tilde{\boldsymbol{x}}^{\rm s},0)$, which can be read off from Eq. (I4). The first term in Eq. (I4) vanishes as $R_b=0$ at QSS, while the remainder is seen to be precisely $c_b(\boldsymbol{x}^{\rm s})$ from Eq. (21). The matrix $\boldsymbol{k}$ in the new variables has elements

$$k_{b'b}=\frac{\partial\tilde{R}_{b'}}{\partial\tilde{x}_b}=\frac{\partial R_{b'}}{\partial x_b}+\sum_s\left[\sum_{b'}(\boldsymbol{J}^{-1})_{bb'}\frac{\partial R_{b'}}{\partial x_s}\right]\frac{\partial R_s}{\partial x_b}=l_{bb'}. \tag{I5}$$

Note that the terms in square brackets are already just dependent on $\boldsymbol{x}^{\rm s}$, so do not contribute to the derivative. The remaining factor in the memory function is, again in the new variables,

$$\frac{\partial\tilde{R}_s}{\partial\tilde{x}_{b'}}=\frac{\partial R_s}{\partial x_{b'}}, \tag{I6}$$

so, overall,

$$\tilde{M}_s^{\rm G}(\boldsymbol{x}^{\rm s},\tau)=\sum_{bb'}\frac{\partial R_s}{\partial x_{b'}}(e^{\boldsymbol{l}(\boldsymbol{x}^{\rm s})\tau})_{bb'}c_b(\boldsymbol{x}^{\rm s})=\sum_{b'}c_{b'}(\boldsymbol{x}^{\rm s})\tilde{f}_{b's}(\boldsymbol{x}^{\rm s},\tau) \tag{I7}$$

with

$$\tilde{f}_{b's}(\boldsymbol{x}^{\rm s},\tau)=\left(e^{\boldsymbol{l}(\boldsymbol{x}^{\rm s})\tau}\right)_{b'b}f^0_{bs}(\boldsymbol{x}^{\rm s}), \tag{I8}$$

where we have used the definition of f^0_{bs} from Eq. (14). Comparing now Eqs. (18) and (I8) shows that the memory approximation Eq. (I7), though derived here from rather different arguments, is quite similar to our expression Eq. (20): The only difference is that the propagation from $\boldsymbol{x}^{\rm s}$ to $\boldsymbol{\phi}_v(\boldsymbol{x}^{\rm s},\tau)$ is absent in $\tilde{f}_{b's}$, which is the analog of our f_{bs}. We show that without the $\boldsymbol{\phi}_v$ propagation, the method can still perform accurately in some situations but breaks down in other settings (Fig. 10).

APPENDIX J: NEURAL TUBE MODEL

We detail the model taken from Ref. [24] used to model ventral neural tube patterning. The equations are as follows:

$$\frac{d}{dt}x_P = \frac{\alpha_P w_{P,p}}{w_{P,p} + (1 + k_{PO}x_O)^2(1 + k_{PN}x_N)^2} - \beta_P x_P, \tag{J1}$$

$$\frac{d}{dt}x_O = \frac{\alpha_O w_{O,p}(1 + k_{O,in}x_{in})}{w_{O,p}(1 + k_{O,in}x_{in}) + (1 + k_{OI}x_I)^2(1 + k_{ON}x_N)^2} - \beta_O x_O, \tag{J2}$$

$$\frac{d}{dt}x_N = \frac{\alpha_N w_{N,p}(1 + k_{N,in}x_{in})}{w_{N,p}(1 + k_{N,in}x_{in}) + (1 + k_{NP}x_P)^2(1 + k_{NO}x_O)^2(1 + k_{NI}x_I)^2} - \beta_N x_N, \tag{J3}$$

$$\frac{d}{dt}x_I = \frac{\alpha_I w_{I,p}}{w_{I,p} + (1 + k_{IO}x_O)^2(1 + k_{IN}x_N)^2} - \beta_I x_I. \tag{J4}$$

The parameters and their meaning in a biological sense are detailed as follows:

Name	Meaning	Value
α_P	Pax6 production rate	2
α_O	Olig2 production rate	2
α_N	Nkx2.2 production rate	2
α_I	Irx3 production rate	2
β_P	Pax6 degradation rate	2
β_O	Olig2 degradation rate	2
β_N	Nkx2.2 degradation rate	2
β_I	Irx3 degradation rate	2
k_{PO}	Olig2 binding to Pax6 DNA	1.9
k_{PN}	Nkx2.2 binding to Pax6 DNA	26.7
k_{ON}	Nkx2.2 binding to Olig2 DNA	60.6
k_{OI}	Irx3 binding to Olig2 DNA	28.4
k_{NP}	Pax6 binding to Nkx2.2 DNA	4.8
k_{NO}	Olig2 binding to Nkx2.2 DNA	27.1
k_{NI}	Irx3 binding to Nkx2.2 DNA	47.1
k_{IO}	Olig2 binding to Irx3 DNA	58.8
k_{IN}	Nkx2.2 binding to Irx3 DNA	76.2
$w_{P,p}$	Polymerase binding to Pax6 DNA	3.84
$w_{O,p}$	Polymerase binding to Olig2 DNA	2.01263
$w_{N,p}$	Polymerase binding to Nkx2.2 DNA	0.572324
$w_{I,p}$	Polymerase binding to Irx3 DNA	18.72
$k_{O,in}$	Gli (Shh signal) binding to Olig2 DNA	180
$k_{N,in}$	Gli (Shh signal) binding to Nkx2.2 DNA	373

The signal input concentration x_{in} is the gradient $e^{-p/0.15}$, which depends on the dorsal-ventral neural tube position p ranging from 0 to 1 as in Ref. [24].
